# How Are Brain Fog Symptoms Related to Diet, Sleep, Mood and Gastrointestinal Health? A Cross-Sectional Study

**DOI:** 10.3390/medicina61020344

**Published:** 2025-02-15

**Authors:** Canan Altinsoy, Derya Dikmen

**Affiliations:** 1 Department of Nutrition and Dietetics, Faculty of Health Sciences, Hacettepe University, 06230 Ankara, Turkey; ddikmen@hacettepe.edu.tr; 2 Department of Nutrition and Dietetics, Faculty of Health Sciences, Recep Tayyip Erdogan University, 53020 Rize, Turkey

**Keywords:** brain fog, mood, COVID-19, gastrointestinal symptoms, mind diet, long COVID

## Abstract

*Background and Objectives*: Brain fog, characterized by cognitive difficulties such as memory impairment, lack of focus, and mental fatigue, is a common symptom reported during recovery from COVID-19, particularly in long COVID cases. This study explores potential triggers such as sleep quality, mood, and gastrointestinal health and examines the link between adherence to the MIND diet and brain fog severity. *Materials and Methods*: A cross-sectional study was conducted between 1 July and 15 December 2022. The questionnaire assessed brain fog symptoms, dietary habits, sleep quality, mood, and gastrointestinal symptoms. Linear regression analysis examined the relationships between brain fog symptoms, demographic factors, sleep quality, MIND diet adherence, and gastrointestinal symptoms. *Results*: Brain Fog Scale (BFS) scores were significantly higher in individuals who had COVID-19 (*p* < 0.05) and even higher in those with reinfection. Women had higher BFS and Brain Fog Severity Score (BFSS), MIND Diet, The Gastrointestinal Symptom Rating Scale (GSRS), Brief Mood Introspection Scale (BMIS) Pleasant-Unpleasant scores (*p* < 0.05). BFS and BFSS were positively correlated with GSRS (*p* < 0.05), while no correlation was found with MIND diet adherence. A negative correlation was observed between BFS and Sleep Quality Scale (SQS) (*p* < 0.05), but this was not significant in regression (*p* = 0.367). GSRS, Pleasant–Unpleasant Dimension, and Arousal–Calm Dimension were significant predictors of BFS (R = 0.599, R^2^ = 0.358, *p* < 0.01). *Conclusions:* This study identifies being female as a risk factor for brain fog symptoms, with women reporting higher BFS and BFSS scores. While sleep quality showed a negative correlation with brain fog symptoms, this relationship was not significant in the regression model, suggesting that other factors, such as mood and gastrointestinal symptoms, may play a more dominant role. However, adherence to the MIND diet showed no significant relationship with brain fog symptoms. These findings suggest that addressing mood and gastrointestinal health may be key to managing brain fog in long COVID.

## 1. Introduction

The approach to the COVID-19 pandemic has undergone changes in the years that followed its onset. Initially, the primary emphasis was on the risk of death during the early stages. However, the pandemic’s psychosocial consequences took center stage, and today, there is increased attention on the persistent symptoms experienced in post-COVID-19 recovery [1]. Individuals infected with COVID-19 report various persistent symptoms after the acute phase of the disease, such as shortness of breath, myalgia, anxiety, extreme fatigue, mood swings, memory problems, sleep disturbances, cough, and attention deficit [2]. COVID-19 causes post-COVID syndrome, characterized by cognitive dysfunction and fatigue. The UK National Institute for Health and Care Excellence defined post-COVID syndrome as “signs and symptoms that may persist 12 weeks after an acute COVID-19 infection and cannot be explained by an alternative diagnosis [3]. When individuals experience fatigue, memory issues, breathing difficulties, or unexplained muscle pain lasting for a duration of up to 2 weeks in mild cases, up to 4 weeks in moderate-severe cases, and up to 6 weeks in critically ill individuals, it is defined as “Prolonged COVID syndrome”. Among the neuropsychiatric symptoms associated with this condition, brain fog has recently gained significant attention [1,4]. Brain fog is a term that encompasses symptoms such as decreased cognitive function and mental acuity, difficulty concentrating and multitasking, and short- and long-term memory loss [5]. The precise etiology of this symptom remains incompletely understood; however, current understanding suggests that aberrant or exaggerated autoimmune and immune responses contribute significantly to its pathogenesis [5,6]. Therefore, implementing strategies to mitigate inflammation may confer beneficial effects.

Nutrition is a key modulator of inflammation, with various dietary patterns and nutrients exhibiting either pro- or anti-inflammatory properties [7]. In recent years, the MIND diet has emerged as one of the most notable dietary patterns for supporting cognitive function [8]. This diet integrates key components of the Mediterranean and DASH diets, aiming to preserve cognitive health by emphasizing the consumption of neuroprotective, antioxidant-rich foods such as leafy green vegetables and berries. The MIND diet prioritizes the intake of fresh fruits and vegetables while promoting brain-healthy foods, including green leafy vegetables, nuts, berries, legumes, whole grains, fish, poultry, olive oil, and moderate amounts of wine. Conversely, it restricts foods considered detrimental to brain health, such as red meat, butter and margarine, cheese, pastries, sweets, and fried or fast foods. What sets the MIND diet apart from other dietary patterns is its specific classification of foods into beneficial and harmful categories, providing a structured approach to optimizing brain health [8,9]. To our knowledge, the relationship between brain fog and dietary patterns has not been studied. In this study, we aim to examine the relationship between the MIND diet, which is prominent for its cognitive health benefits, and brain fog symptoms.

Sleep is a natural and reversible state marked by decreased responsiveness to external stimuli and relative inactivity, accompanied by a loss of consciousness. It occurs in regular cycles and is regulated by homeostatic mechanisms; when sleep is lost or delayed, individuals often experience extended periods of sleep afterward, which can lead to various physiological and psychological disruptions [10]. It has been shown that COVID-19 infection negatively affects sleep in patients either through a direct impact on the central nervous system (CNS) or as a result of other COVID-19-related symptoms, such as fever, headache, dyspnea, myalgia, throat pain, cough, gastrointestinal disturbances, fatigue, anxiety, and depression, all of which can contribute to poor sleep quality [11]. Moreover, many individuals report experiencing difficulty initiating and maintaining sleep, insomnia, poor sleep quality, and excessive sleepiness even months after recovering from the initial infection [12]. Sleep disturbances in long COVID have been highlighted in the literature [13,14,15], emphasizing the need for further research to explore the underlying mechanisms linking sleep quality to cognitive dysfunction, particularly brain fog symptoms.

A strong relationship between mood and cognitive functions has been reported, and controlling mood symptoms may contribute to improved cognitive functions and better functional outcomes [16,17]. Studies have observed that mood changes are among the most frequently reported symptoms of long COVID [18,19,20,21,22,23]. However, evidence regarding the potential role of mood changes in the development of brain fog remains limited [24]. Therefore, this study aims to explore the possible influence of mood on brain fog.

The gut–brain axis (GBA) comprises a bidirectional communication network between the central and enteric nervous systems, linking the brain’s emotional and cognitive centers with peripheral gut functions [25]. In recent years, growing evidence has highlighted the critical role of this interaction in maintaining cognitive health [26,27]. While numerous studies have reported an increase in gastrointestinal symptoms among individuals with long COVID [28,29], research specifically examining their role in brain fog remains limited. Therefore, this study aims to investigate the relationship between gastrointestinal symptoms and brain fog symptoms, shedding light on the potential role of gut health in brain fog.

Given the increasing prevalence of persistent cognitive symptoms after COVID-19, it is essential to identify modifiable lifestyle factors that may mitigate brain fog [1,4]. While the MIND diet is well-documented for its neuroprotective properties, its potential role in alleviating brain fog remains unexplored [8,9]. Additionally, although sleep disturbances, mood changes, and gastrointestinal symptoms have been linked to cognitive function, their specific associations with brain fog have not been thoroughly investigated [13,18,28]. To address these gaps, our study aimed to take a holistic approach, integrating multiple lifestyle and physiological factors to provide a comprehensive understanding of brain fog symptoms.

## 2. Materials and Methods

This cross-sectional study was conducted in Türkiye between 1 July 2022, and 15 December 2022 (in accordance with the STROBE checklist). All individuals meeting the eligibility criteria were invited to participate; the participation was not restricted to specific geographic regions of the country. Recruitment was conducted through social media announcements, and participants were encouraged to share the survey link with their acquaintances. The study followed the guidelines set forth in the Declaration of Helsinki and all protocols received approval from the Non-Interventional Clinical Research Ethics Committee of Hacettepe University (Project Number: GO 22/467 Decision number: 2022/10-39).

Data were collected via Google Forms. Before the survey, participants were provided with detailed information about the study’s aim, voluntary participation, and data anonymity. Additionally, they were informed that they could withdraw from the study at any time. To ensure informed consent, only participants who selected the option ’I agree to participate in the study’ were able to proceed with the questionnaire.

### 2.1. Participants

A sample size of 300 individuals with 95% confidence was calculated using G*Power 3.1.9.7 software, considering a 7.2% incidence rate of brain fog as reported in a previous study evaluating the impact of brain fog on COVID-19-infected individuals [1].

**Figure 1:**
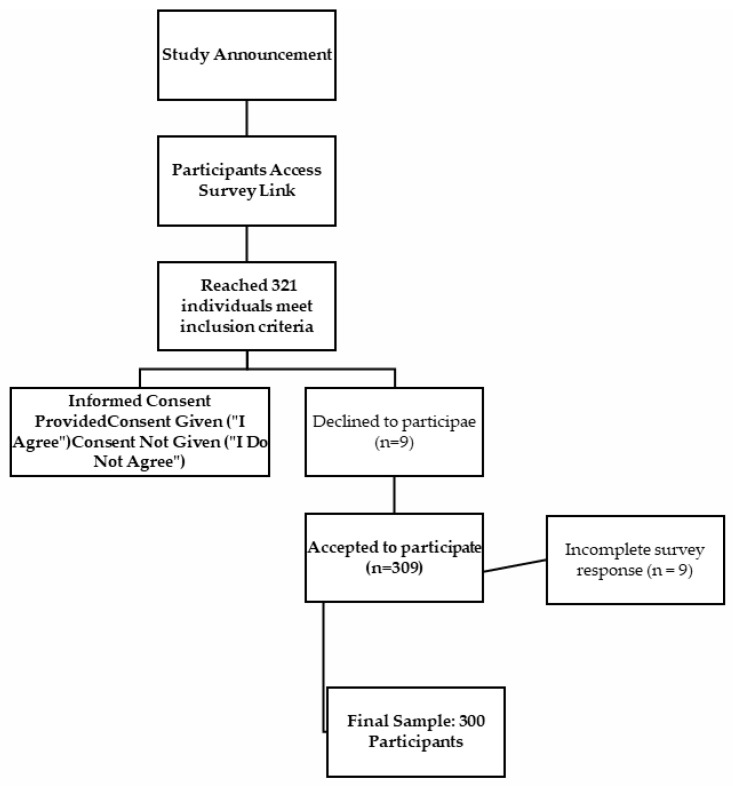
Participant recruitment flowchart.

Inclusion criteria encompassed adult males and females between the ages of 19 and 64 who willingly consented to participate. Participants were required to have no dietary restrictions due to health conditions, no diagnosed chronic diseases affecting dietary intake, and no ongoing medical treatments that could influence gastrointestinal function or cognitive performance. Exclusion criteria involved individuals below 19 or above 65 years of age, those who followed specific diets for health reasons (including vegans and vegetarians), individuals with food allergies and intolerances (such as celiac disease and lactose intolerance), individuals following dietary plans due to chronic diseases, individuals using antidepressants, and individuals receiving medical treatment for gastrointestinal and sleep disorders (Figure 1).

### 2.2. Questionnaire

The data were gathered through an online questionnaire using Google Forms. The questionnaire included demographic questions (age, gender, body weight, and height) and scales to assess brain fog, diet quality, sleep, mood, and gastrointestinal symptoms, which are detailed below.

Brain Fog Scale (BFS): This was developed by Debowska et al. to describe and psychometrically evaluate the symptoms of brain fog [30]. A validity and reliability study for the Turkish population was conducted by Bas et al. [31]. Cronbach’s alpha coefficient was 0.966 in that study. The scale is scored on a 5-point Likert scale ranging from “never” to “always”. The BFS is a valid scale with good psychometric properties for assessing brain fog in clinical and research settings. In the current study, Cronbach’s alpha coefficient was 0.967.

Brain Fog Severity Score (BFSS): The Brain Fog Severity Score was used to assess brain fog severity. Individuals rated the severity of brain fog they experienced from 0 to 100 in 10-point intervals. Higher scores on the scale indicate increased brain fog [8].

MIND Diet Score (Mediterranean-DASH Intervention for Neurodegenerative Delay-MIND Diet): This scale was constructed based on the nutrient components of the Mediterranean and Dietary Approaches to Stop Hypertension (DASH) diets, which are beneficial for dementia and cognitive decline. When determining the MIND diet score, 0, 0.5, or 1 point is given according to the amount and portion consumed. The total score is obtained by summing the scores from these 15 foods. High MIND diet scores are associated with high compliance with the MIND diet and a slowing of cognitive decline [32]. In this study, Cronbach’s alpha coefficient was 0.790. 

Single-Item Sleep Quality Scale (SQS): Individuals’ sleep quality was obtained by subjectively evaluating their sleep in the last seven days in a single item. The scale developed by Snyder et al. shows a good correlation with the Pittsburgh Sleep Quality Index score [33].

Brief Mood Introspection Scale (BMIS): The scale developed by Mayer and Gaschke (1988) consists of 16 items (such as happy, sad, tired, interested, etc.) that assess mood state. The scale has four sub-dimensions: pleasant–unpleasant, emotionally arousal–calm, positive–tired, and negative–relaxed. The score of each sub-dimension covers a different number of items. For example, the “pleasant–unpleasant” sub-dimension covers all 16 items, while the sub-dimensions of “arousal–calm”, “positive–tired”, and “negative–relaxed” cover 12, 7, and 6 items, respectively [34]. Cronbach’s alpha coefficient was 0.745 for this metric in present study.

The Gastrointestinal Symptom Rating Scale (GSRS): This is a 15-item instrument that employs a 7-point Likert-type response format, ranging from “no discomfort” to “very severe discomfort”. Through factor analysis, the 15 items of the GSRS are classified into five sub-dimensions: Abdominal Pain, Reflux, Diarrhea, Indigestion, And Constipation. The Gastrointestinal Health Index (GHI) assesses an individual’s gastrointestinal symptomatology over the past week. Higher scores on the GHI indicate more severe symptoms. The scale, originally developed by Revicki et al. in 1998, was adapted and validated in Turkish by Turan et al. in 2017 [35,36]. In the current study, the Cronbach’s alpha coefficient was 0.919.

### 2.3. Statistical Analysis

Data analysis was performed using IBM SPSS software (version 22.00). Cronbach’s alpha analysis of the scales was calculated to determine their suitability for use in our sample. In the analysis of the data according to brain fog symptoms, the t test was used for comparisons of two independent variables, one-way analysis of variance was used for multiple comparisons (Dunnet and T3 for post hoc tests), and the Kruskal–Wallis test was used for non-normally distributed data. Cohen’s d was calculated to assess the effect size for significant group differences. Linear regression analysis was applied to understand the brain fog symptoms according to age, gender, Body Mass Index (BMI), years of employment, and scores of SQS, MIND diet, GSRS, and BMIS. Statistical significance was accepted as *p* < 0.05.

## 3. Results

Table 1 shows that the mean ages of the women and men were 31.84 ± 10.85 years and 36.25 ± 10.22 years, respectively. Further, 54% of women were normal weight, while 45.3% of men were overweight. COVID-19 affected 68.6% of women and 51.6% of men, while 17.4% of women and 14.6% of men had it more than once.

Table 2 presents the mean and standard deviation values for the BFS, BFSS, and MIND diet scores based on COVID-19 infection status, reinfection, and disease severity. Individuals who had been infected with COVID-19 had significantly higher BFS scores than those who had not (*p* = 0.024). However, no significant differences were found in BFSS (*p* = 0.212) and MIND diet scores (*p* = 0.266) between infected and non-infected individuals. Participants who had COVID-19 more than once had significantly higher BFS scores than those who had it only once (*p* = 0.024). BFSSs were marginally higher in those with reinfection (*p* = 0.088), while MIND diet scores did not show a significant difference (*p* = 0.334). BFS scores tended to be higher in participants who reported severe symptoms at home and those who were hospitalized, but the difference did not reach statistical significance (*p* = 0.055). BFSSs were significantly different among severity groups (*p* = 0.020), with those experiencing severe symptoms at home scoring higher than those with mild symptoms (*p* = 0.020, 2 > 1). MIND diet score did not show a significant difference based on disease severity (*p* = 0.869).

Table 3 presents the mean and standard deviation values for the BFS, BFSS, SQS, MIND diet score, GSRS, and BMIS, stratified by gender and comparison groups (C+ vs. C−). Significant differences were found in BFS (*p* = 0.001), BFSS (*p* = 0.000), MIND diet score (*p* = 0.001), GSRS (*p* = 0.05) and BMIS Pleasant–Unpleasant (*p* = 0.021) scores between males and females. BFS (*p* = 0.024) scores showed significant differences between the C+ and C− groups.

Table 4 shows that the BFS scores and SQS scores were negatively correlated (*p* < 0.001), and the BFS scores and Abdominal Pain Dimension, Reflux Dimension, Diarrhea Dimension, Indigestion Dimension, Constipation Dimension, and GSRS scores were positively correlated (*p* < 0.001). Age, BFS, Abdominal Pain Dimension, Diarrhea Dimension, and Pleasant–Unpleasant Dimension scores were negatively correlated (*p* < 0.001). The BMI and MIND diet score, Pleasant–Unpleasant Dimension, BMIS Arousal–Calm Dimension, and BMIS Negative–Relaxed Dimension scores were negatively correlated (*p* < 0.001).

Table 5 shows that GSRS, Pleasant–Unpleasant Dimension, and Arousal–Calm Dimension variables are significant predictors of brain fog symptoms ((R = 0.599, R^2^ = 0.358, *p* < 0.001).

## 4. Discussion

Brain fog is one of the most commonly reported symptoms after a COVID-19 infection [37,38,39,40,41,42,43,44,45,46,47]. Studies have also reported that women experience higher levels of brain fog symptoms than men [48,49,50,51,52]. Our study corroborates these findings, demonstrating that individuals with a history of COVID-19 infection exhibited significantly higher mean BFS scores compared to those without prior infection. Furthermore, women were more likely to experience brain fog symptoms. This pattern aligns with previous research conducted in Iran, which demonstrated a higher prevalence of brain fog among women following COVID-19 [1]. Similarly, Sudre et al. reported that long COVID had a greater impact on women than men [53]. One possible explanation for this difference is genetic and hormonal factors. Women tend to have a stronger immune response and heightened T-cell activation, which, while providing survival advantages, may also contribute to prolonged inflammation, potentially making them more susceptible to brain fog [54,55]. Beyond biological mechanisms, psychosocial factors may also contribute to these gender differences. Sykes et al. suggested that not only the direct effects of COVID-19 infection but also the lifestyle changes induced by the pandemic could play a role in the prolonged cognitive symptoms observed in women. Increased levels of depression, reduced physical activity, and heightened caregiving responsibilities during the pandemic may have exacerbated the persistence of brain fog [2,56]. Additionally, women generally tend to seek medical care more frequently than men, which could partly explain the higher reported prevalence of brain fog in this population [46,57]. Further research is needed to disentangle the interplay between biological, psychological, and social determinants contributing to gender disparities in post-COVID cognitive symptoms. Understanding these mechanisms could help in developing targeted interventions to mitigate long COVID effects and improve cognitive health outcomes.

The findings of this study revealed a significant negative correlation between sleep quality (SQS) and brain fog scores (BFS). However, regression analysis indicated that this relationship became non-significant when controlling for other variables (mood, diet, gastrointestinal symptoms). This suggests that the impact of sleep quality on brain fog may be mediated by other factors, such as gastrointestinal symptoms or mood disturbances. Given the well-established role of the gut–brain axis and neuroinflammation in cognitive function, these pathways warrant further exploration. While our findings partially align with previous research, they also introduce nuances that merit discussion. For instance, Robinson et al. demonstrated that sleep deprivation is one of the most potent triggers of brain fog [47]. Similarly, Orfei et al. observed elevated brain fog scores in individuals with sleep disturbances [52], and Azcue et al. reported a strong association between post-COVID-19 brain fog and poor sleep quality [43]. However, unlike these studies, our results suggest that the relationship between sleep quality and brain fog may be more complex and potentially influenced by indirect mechanisms rather than a direct causal link. These differences might stem from variations in study design, sample characteristics, or the inclusion of additional confounding variables. A more comprehensive approach incorporating both subjective and objective sleep measures (e.g., polysomnography, actigraphy) may also help to clarify the underlying mechanisms and contextualize our findings within the broader literature.

The role of the gastrointestinal system in the pathophysiology of neuropsychiatric and neurophysiological disorders has been well established [58,59]. SARS-CoV-2 induces systemic inflammation, which disrupts the gut–brain axis [60,61]. Notably, COVID-19 has been associated with intestinal damage, and gastrointestinal symptoms are as prevalent as respiratory symptoms in affected individuals [61]. In the present study, a positive correlation was found between individuals’ GSRS scores and their BFS scores, indicating that gastrointestinal symptoms may contribute to the severity of brain fog. Brain fog was observed in various autoimmune and inflammatory diseases targeting the gastrointestinal system, such as celiac disease and Crohn’s disease, even before the COVID-19 pandemic [62,63]. Furthermore, a case study by Anderson and Pitsinger demonstrated that dietary changes, such as eliminating gluten-containing foods, led to a reduction in both brain fog and gastrointestinal symptoms [64]. Given these findings, it is plausible that neurological issues may stem from intestinal inflammation and a compromised gut barrier, often referred to as “leaky gut”, triggered by various factors. Consequently, interventions targeting gastrointestinal health may play a crucial role in alleviating brain fog symptoms.

Our findings align with previous research suggesting a strong link between psychological factors and brain fog symptoms following COVID-19. Krishnan et al. reported that psychological symptoms, particularly mood disturbances, may contribute to brain fog. Similarly, our findings suggest that mood plays a crucial role in the development and severity of brain fog symptoms [24]. Long COVID syndrome has been shown to impair daily activities and functional abilities [42], negatively impact emotional and mental health [41], reduce work productivity [65], diminish functional independence, and lower overall quality of life [44]. Given these widespread effects, the appropriate psychological assessment and management of mental health conditions associated with or exacerbated by persistent post-COVID-19 symptoms are essential for improving both cognitive and overall well-being.

This study did not find a significant relationship between MIND diet scores and brain fog. To the best of our knowledge, no previous studies have specifically investigated the association between MIND diet adherence and brain fog symptoms. However, Krishnan’s study highlights the role of nutrition in managing brain fog and emphasizes the importance of including a dietitian in the multidisciplinary team for effective treatment [24]. Since brain fog is commonly reported after COVID-19, long-term follow-up studies are needed to assess whether dietary patterns influence cognitive recovery in affected individuals.

This study has several strengths, including its relevance to the timely issue of brain fog in long COVID, its comprehensive approach in assessing multiple potential triggers such as sleep quality, mood, and gastrointestinal health, and the identification of important predictors like mood and gastrointestinal health. Additionally, it highlights gender differences in brain fog symptoms, providing valuable insights into potential gender-specific interventions. However, the study’s cross-sectional design limits its ability to establish causal relationships. Another limitation is the reliance on self-reported data collected through online questionnaires. While self-report measures are commonly used in research, they are prone to biases, such as social desirability and recall bias, which could affect the accuracy of the responses. Furthermore, online data collection introduces additional limitations, including variability in participant engagement and a lack of control over the testing environment. Additionally, one limitation of this study is that the sample was obtained through social media. While this method allowed for broader participation, it may have introduced selection bias and limited the generalizability of the findings. Future studies using more diverse and structured sampling methods could provide more representative results. Additionally, the online administration of the study may have influenced the accuracy of responses, as participants may have interpreted questions differently or been subject to external distractions, further impacting the reliability of the data. These limitations should be taken into account when interpreting the results. Future studies should consider incorporating objective measures, such as 24 h dietary recalls, food diaries, biomarkers, actigraphy, or polysomnography, to provide a more precise and comprehensive evaluation of dietary intake, adherence, and sleep quality, which would enhance the validity and reliability of the findings. To better understand the relationship between gut health and brain fog, microbiota studies could be conducted. These studies could explore how gut microbiota composition and function influence cognitive symptoms, including brain fog, shedding light on potential mechanisms linking gastrointestinal health with cognitive function. To address these limitations, future research should employ longitudinal designs, incorporate objective measurements, and investigate larger populations to enhance the generalizability and accuracy of the findings.

## 5. Conclusions

This study highlights the multifaceted nature of brain fog symptoms, particularly in individuals recovering from COVID-19. Individuals who had been infected with COVID-19 had significantly higher BFS scores than those who had not, and this effect was even more pronounced in those who experienced reinfection. These findings suggest that repeated exposure to SARS-CoV-2 may exacerbate the cognitive symptoms associated with brain fog. While BFS scores were higher in individuals who reported severe symptoms at home and in those who were hospitalized, the difference did not reach statistical significance. However, BFSSs were significantly higher in participants with severe symptoms at home compared to those with mild symptoms, suggesting that disease severity may influence the intensity of brain fog symptoms. Our findings indicate that women experience greater brain fog severity, suggesting a potential gender-related vulnerability. While sleep quality showed a negative correlation with brain fog symptoms, this relationship was not significant in the regression model, implying that other factors, such as mood and gastrointestinal symptoms, may exert a stronger influence. Notably, gastrointestinal symptom severity emerged as a significant predictor of brain fog, reinforcing the role of gut–brain interactions in cognitive function. Furthermore, adherence to the MIND diet did not show a significant association with brain fog symptoms, indicating that dietary patterns alone may not be a determining factor in symptom severity. Given these findings, interventions targeting mood regulation and gastrointestinal health may be more effective in alleviating brain fog symptoms in long-COVID patients. Future longitudinal studies are needed to further explore the causal relationships between these factors and to develop targeted strategies for managing post-COVID cognitive impairment.

## Figures and Tables

**Table 1:** Descriptive characteristics of the participants.

	Female (*n* = 172)		Male (*n* = 128)	
	*n*	%	*n*	%
Age (years)				
Min–Max	19–62		21–62	
mean ± SD	31.84 ± 10.85		36.25 ± 10.22	
Education				
Primary–high school	25	14.5	24	18.75
University	109	63.3	69	53.9
Master’s/PhD	38	22	35	27.3
Occupation				
Worker	2	1.1	25	19.5
Health sector	60	34.9	14	10.9
Education and research sectors	57	33.1	28	21.8
Civil servant/desk-based employee	31	18	48	37.5
Housewife/not working/retired	22	12.8	13	10.1
Working year				
Min–Max	1–37		1–42	
mean ± SD	10.27 ± 10.11		12.91 ± 9.98	
Smoking				
Yes	30	17.4	44	34.3
No	142	83.55	84	65.7
Alcohol				
Yes	25	14.5	39	30.4
No	147	85.5	89	69.6
Physical activity an hour three times a week				
Yes	61	35.4	68	53.1
No	111	64.6	60	46.9
Infected with COVID-19				
Yes	118	68.6	66	51.6
No	54	31.4	62	48.4
Infected with COVID-19 more than once				
Yes	30	17.4	19	14.6
No	142	82.6	109	85.4
BMI category				
Underweight	14	8.1	1	0.8
Normal weight	93	54.0	50	39
Overweight	43	25	58	45.3
Obese	22	12.8	19	14.8
BMI (kg/m^2^)				
Min–Max	15.57–37.65		15.57–37.65	
mean ± SD	24.02 ± 4.74		26.18 ± 3.76	

BMI = Body Mass Index, SD = standard deviation.

**Table 2:** Comparison of brain fog symptoms, brain fog severity, and MIND diet score based on COVID-19 status, disease severity and gender.

		BFS		BFSS		MIND Diet Score		TOTAL		
		Female	Male	Female	Male	Female	Male	BFS	BFSS	MIND Diet Score
		X ± S.D.	X ± S.D.	X ± S.D.	X ± S.D.	X ± S.D.	X ± S.D.	X ± s.s.	X ± s.s.	X ± s.s.
Infected with COVID-19	Yes	58.43 ± 21.899	46.94 ± 20.744	5.41 ± 2.549	3.96 ± 2.888	6.35 ± 1.671	5.74 ± 1.615	54.27 ± 22.135	4.88 ± 2.761	6.13 ± 1.673
	No	51.93 ± 17.694	46.47 ± 17.221	4.83 ± 2.601	4.13 ± 2.607	6.83 ± 1.546	5.92 ± 2.035	49.13 ± 17.595	4.48 ± 2.616	6.36 ± 1.863
		***p* = 0.035**	*p* = 0.888	*p* = 0.161	*p* = 0.587	*p* = 0.0717	*p* = 0.717	***p* = 0.024**	*p* = 0.212	*p* = 0.266
Having COVID-19 more than once	Yes	66.33 ± 22.093	45.74 ± 17.026	6.10 ± 2.203	4.11 ± 2.807	6.22 ± 1.541	5.66 ± 1.434	58.35 ± 22.503	5.33 ± 2.617	6.00 ± 1.510
	No	54.26 ± 19.983	46.88 ± 19.467	5.03 ± 2.613	4.03 ± 2.754	6.57 ± 1.661	5.85 ± 1.883	51.11 ± 20.063	4.61 ± 2.715	6.26 ± 1.791
		***p* = 0.003**	*p* = 0.853	***p* = 0.038**	*p* = 0.884	*p* = 0.287	*p* = 0.532	***p* = 0.024**	*p* = 0.088	*p* = 0.334
Disease process	1. At home, I got through it with mild symptoms.	55.93 ± 21.432	45.85 ± 20.542	4.97 ± 2.380	3.98 ± 2.886	6.50 ± 1.777	5.84 ± 1.753	51.48 ± 21.550	4.53 ± 2.650	6.21 ± 1.790
	2. At home, I had severe symptoms.	62.54 ± 22.306	50.21 ± 20.622	6.06 ± 2.637	4.14 ± 3.035	6.20 ± 1.519	5.68 ± 1.395	59.92 ± 22.388	5.64 ± 2.819	6.09 ± 1.498
	3. At the hospital, I was under observation in the ward.	51.00 ± 31.113	69.50 ± 2.121	4.50 ± 3.536	8.50 ± 2.121	5.50 ± 1.414	6.25 ± 1.061	60.25 ± 20.934	6.50 ± 3.317	5.88 ± 1.109
		*p* = 0.257	*p* = 0.191	*p* = 0.062	*p* = 0.159	*p* = 0.472	*p* = 0.807	KW = 5.811	KW = 7.805	KW = 0.280
								*p* = 0.055	***p* = 0.020**	*p* = 0.869
								-	2 > 1	-

BFS = Brain Fog Scale; BFSS = Brain Fog Severity Score; KW = Kruskal–Wallis H test. A bolded *p*-value indicates a statistically significant difference (*p* < 0.05).

**Table 3:** Comparison of scores on BFS, BFSS, SQS, MIND diet, GSRS, and BMIS by COVID status and gender.

Variable	Total (Mean ± SD)	C+ (Mean ± SD)	C− (Mean ± SD)	*p*- (Gender)	*p*- (C+ vs. C−)
BFS	52.26 ± 20.60	54.27 ± 22.14	49.13 ± 17.60	**0.001**	**0.024**
				(Cohen’s d = 0.28)	(Cohen’s d = 0.26)
Female	56.28 ± 20.78	58.43 ± 21.90	51.93 ± 17.69		
Male	46.72 ± 19.07	46.94 ± 20.74	46.47 ± 17.22		
BFSS	4.72 ± 2.71	4.88 ± 2.76	4.48 ± 2.62	**0.000**	0.212
				(Cohen’s d = 0.44)	
Female	5.21 ± 2.57	5.41 ± 2.55	4.83 ± 2.60		
Male	4.04 ± 2.75	3.96 ± 2.89	4.13 ± 2.61		
SQS	6.01 ± 2.27	5.94 ± 2.18	6.11 ± 2.40	0.300	0.527
Female	5.89 ± 2.21	5.78 ± 2.16	6.12 ± 2.32		
Male	6.17 ± 2.34	6.23 ± 2.20	6.10 ± 2.49		
MIND Diet Score	6.22 ± 1.75	6.13 ± 1.67	6.36 ± 1.86	**0.001**	0.266
				(Cohen’s d = 0.39)	
Female	6.51 ± 1.64	6.35 ± 1.67	6.83 ± 1.55		
Male	5.83 ± 1.82	5.74 ± 1.62	5.92 ± 2.04		
GSRS	38.22 ± 18.98	39.81 ± 19.17	35.77 ± 18.50	**0.05**	0.069
				(Cohen’s d = 0.21)	
Female	40.80 ± 18.68	40.92 ± 18.91	40.56 ± 18.37		
Male	34.64 ± 18.89	37.84 ± 19.62	31.13 ± 17.56		
BMIS Pleasant–Unpleasant	40.39 ± 5.27	40.55 ± 4.91	40.12 ± 5.79	**0.021**	0.485
				(Cohen’s d = 0.28)	
Female	41.01 ± 4.38	40.76 ± 4.28	41.51 ± 4.55		
Male	39.53 ± 6.21	40.19 ± 5.88	38.81 ± 6.52		
BMIS Arousal–Calm	30.40 ± 4.15	30.40 ± 3.97	30.39 ± 4.43	0.295	0.975
Female	30.62 ± 3.51	30.36 ± 3.47	31.15 ± 3.54		
Male	30.09 ± 4.90	30.49 ± 4.75	29.66 ± 5.05		
BMIS Positive–Tired	17.76 ± 2.60	17.81 ± 2.46	17.69 ± 2.82	0.414	0.674
Female	17.87 ± 2.28	17.71 ± 2.21	18.20 ± 2.40		
Male	17.62 ± 2.99	18.00 ± 2.85	17.19 ± 3.10		
BMIS Negative–Relaxed	14.91 ± 2.24	14.95 ± 2.22	14.86 ± 2.27	0.077	0.738
Female	15.11 ± 1.99	15.03 ± 2.04	15.29 ± 1.88		
Male	14.64 ± 2.53	14.81 ± 2.52	14.45 ± 2.55		

BFS = Brain Fogginess Symptom Questionnaire; BMIS = Brief Mood Introspection Scale; GSRS = Gastrointestinal Symptom Rating Scale; SQS = Single Item Sleep Quality Scale; BFSS = Brain Fog Severity Score; C+ = infected with COVID-19; C− = not infected with COVID-19. A bolded *p*-value indicates a statistically significant difference (*p* < 0.05).

**Table 4:** Correlations of BFS, BFSS, SQS, MIND diet score, GSRS, BMIS, age, BMI.

	1	2	3	4	5	6	7	8	9	10	11	12	13	14	15
1-BFS	1														
2-BFSS	0.611 **	1													
3-SQS	−0.314 **	−0.237 **	1												
4-MIND Diet Score	0.007	−0.078	0.070	1											
5-Abdominal Pain	0.360 **	0.341 **	−0.225 **	−0.072	1										
6-Reflux	0.282 **	0.264 **	−0.156 **	−0.103	0.757 **	1									
7-Diarrhea	0.273 **	0.296 **	−0.124 *	−0.058	0.565 **	0.519 **	1								
8-Indigestion	0.294 **	0.273 **	−0.173 **	−0.057	0.712 **	0.657 **	0.614 **	1							
9-Constipation	0.278 **	0.299 **	−0.178 **	−0.022	0.508 **	0.432 **	0.482 **	0.584 **	1	0.754 **					
10-GSRS	0.352 **	0.355 **	−0.201 **	−0.071	0.849 **	0.795 **	0.773 **	0.897 **	0.754 **	1					
11–Pleasant–unpleasant	0.176 **	0.043	−0.107	0.075	0.036	0.012	−0.104	−0.016	−0.040	−0.032	1				
12–Arousal–calm	0.016	−0.067	0.013	0.087	−0.078	−0.071	−0.165 **	−0.100	−0.132 *	−0.134 *	0.946 **	1			
13–Positive–tired	0.032	−0.089	0.022	0.066	−0.023	−0.029	−0.130 *	−0.057	−0.113 *	−0.092	0.885 **	0.897 **	1		
14–Negative–relaxed	0.126 *	0.060	−0.093	0.078	−0.014	−0.018	−0.102	−0.044	−0.048	−0.054	0.874 **	0.885 **	0.636 **	1	
15-Age	−0.189 **	−0.065	0.157 **	−0.045	−0.224 **	−0.065	−0.190 **	−0.034	−0.021	−0.109	−0.155 **	−0.079	−0.065	−0.105	1
16-BMI	−0.075	−0.034	0.016	−0.173 **	0.022	0.092	0.020	0.077	0.022	0.068	−0.139 *	−0.127 *	−0.094	−0.125 *	0.506 **

(*) *p* < 0.05 (**) *p* < 0.001.

**Table 5:** Linear regression analysis results for BFS.

Variable	B	Standard Error	Beta	T	*p*
Constant	35.073	14.563		2.408	0.017
Age	0.031	0.260	0.016	0.121	0.904
Gender	−3.372	2.292	−0.085	−1.471	0.143
BMI	−0.358	0.286	−0.079	−1.253	0.211
Working Year	−0.068	0.251	−0.035	−0.270	0.787
SQS	−0.460	0.509	−0.052	−0.905	0.367
MIND Diet Score	0.011	0.608	0.001	0.018	0.986
GSRS	0.221	0.063	0.208	3.482	0.001
BMIS Pleasant–unpleasant	3.944	0.995	1.041	3.964	0.000
BMIS Arousal–calm	−7.654	1.705	−1.597	−4.490	0.000
BMIS Positive–tired	2.627	2.143	0.349	1.226	0.221
BMIS Negative–relaxed	3.453	2.303	0.385	1.499	0.135
R = 0.599	R^2^ = 0.358				
F(11.226) = 11.467 *p* = 0.000					

BFS = Brain Fogginess Symptom Questionnaire; BMI = Body Mass Index; SQS = Single Item Sleep Quality Scale; GSRS = Gastrointestinal Symptom Rating Scale; BMIS = Brief Mood Introspection Scale.

## Data Availability

The data can be obtained from the corresponding author upon request.
